# Temporal patterns in the soundscape of a Norwegian gateway to the Arctic

**DOI:** 10.1038/s41598-022-11183-y

**Published:** 2022-05-10

**Authors:** A. S. Aniceto, E. L. Ferguson, G. Pedersen, A. Tarroux, R. Primicerio

**Affiliations:** 1 Department of Fisheries and Bioeconomics, Arctic University of Norway, Tromsø, Norway; 2 Ocean Science Analytics, San Diego, CA USA; 3 Department of Marine Ecosystem Acoustics, Institute of Marine Research, 1870 Bergen, Norway; 4 Fram Centre – High North Research Centre for Climate and Environment, Norwegian Institute for Nature Research, 9296 Tromsø, Norway; 5 Fram Centre – High North Research Centre for Climate and Environment, Institute of Marine Research, 9296 Tromsø, Norway

**Keywords:** Ecology, Environmental sciences, Physics, Ocean sciences, Marine biology, Behavioural ecology, Animal behaviour

## Abstract

As an Arctic gateway, the Norwegian Sea sustains a rich diversity of seasonal and resident species of soniferous animals, vulnerable to the effects of climate change and anthropogenic activities. We show the occurrence of seasonal patterns of acoustic signals in a small canyon off Northern Norway, and investigate cetacean vocal behavior, human-made noise, and climatic contributions to underwater sound between January and May 2018. Mostly median sound levels ranged between 68.3 and 96.31 dB re 1 μPa^2^ across 1/3 octave bands (13 Hz–16 kHz), with peaks in February and March. Frequencies under 2 kHz were dominated by sounds from baleen whales with highest rates of occurrence during winter and early spring. During late-spring non-biological sounds were predominant at higher frequencies that were linked mainly to ship traffic. Seismic pulses were also recorded during spring. We observed a significant effect of wind speed and ship sailing time on received sound levels across multiple distance ranges. Our results provide a new assessment of high-latitude continental soundscapes in the East Atlantic Ocean, useful for management strategies in areas where anthropogenic pressure is increasing. Based on the current status of the local soundscape, we propose considerations for acoustic monitoring to be included in future management plans.

## Introduction

The acoustic environment is a key feature of marine habitats^1–4^ and plays an important role in ecosystem functioning as marine animals depend on sound for communication^5–7^, foraging^8–10^, reproduction^11^, predator detection^12^, and navigation^13,14^. All sounds, including biophony (representing biological activity, such as animal vocalizations), geophony (geophysical and meteorological events), and anthrophony (anthropogenic activities), contribute to a given landscape to create unique acoustic patterns across a variety of spatial and temporal scales^15^. Soundscapes not only represent a holistic view of the current state of the environment^16–18^, but can also show how it evolves over time. Thus, soundscapes are not static features of the environment and vary spatially and temporally^19^.

Through the use of fixed and mobile passive acoustic listening systems, soundscape analyses have been successfully used to evaluate patterns of biodiversity in relation to environmental disturbances^2,3,20^, animal movement^21,22^, and species’ temporal variation across multiple scales^17,23,24^. With increasing marine background noise over the past 50 years due to the growth of anthropogenic activities^17,25,26^, several studies have demonstrated the effects of noise pollution on the communication, behavior and physiological state of fish, marine mammals and crustaceans^27–30^. Noise overlapping with biological sounds can cause different responses in animal vocalizations, such as raising the intensity^31,32^ or changing the frequency of the vocalization^33,34^. Furthermore, overlap in frequencies between anthropogenic sounds and vocalizing or listening animals might alter many vital functions, such as echolocation and the detection distance of a predator or conspecific^17,35^. The rise in the quantity and intensity of stressors, combined with a continuously growing body of knowledge on their detrimental effects, demand appropriate management actions that consider the acoustic environment as a vital component of marine ecosystems.

Several studies have highlighted Arctic regions as areas of priority for monitoring marine soundscapes^3,23,36^. Transition zones into the high-Arctic where migratory species tend to gather seasonally are also expected to experience changes in population dynamics and other ecological processes. These zones support processes of climatic and ecological importance, in that they promote thermohaline circulation^37^, and act as routes^38,39^ and foraging grounds for migratory^40–42^ and resident species^43–45^. They are also subjected to many direct anthropogenic stressors and can provide the first indications of disturbance that may be used for understanding the effects of these stressors in polar regions.

During the last decade, the highly productive Norwegian Sea has experienced increased anthropogenic activity (fishing activity, seismic surveys, oil and gas extraction)^46^, increased water temperature^47,48^, a shift in timing of the phytoplankton blooms^49^ and a decrease in zooplankton biomass^50^. The Norwegian Sea hosts large oceanic fish stocks, and represents an important feeding ground for many fish predators, as well as both spawning and feeding habitats for many marine species^51^. Several cetacean species are known to occur in these waters, including the globally vulnerable fin^52^ (*Balaenoptera physalus*) and sperm whales (*Physeter macrocephalus*), as well as humpback (*Megaptera novaeangliae*), minke (*Balaenoptera acutorostrata*), pilot (*Globicephala melas*), and killer whales (*Orcinus orca*), and some species of dolphins (e.g., white-beaked dolphin, *Lagenorhynchus albirostris*)^53^. Within the Lofoten-Barents Sea’s management region, Lofoten–Vesterålen (LoVe) is internationally known as the most important area for commercial fishing in Norway^54^ and a hotspot for eco-tourism. From late autumn to late winter, this area is occupied by humpback whales foraging on Norwegian spring-spawning herring^41^. The same period of the year also represents the peak of killer whale sightings^41^, though these can also be observed year-round in Norwegian waters. The only other large odontocete that can be observed in all seasons in this area is the sperm whale, as the males use deep-water canyons for foraging^55^. Yet, little is known about the seasonal occurrence and distribution of these and other cetacean species outside the summer months mainly due to logistic constraints for marine mammal detection during periods of low light (e.g., polar night).

Overcoming data gaps in temporal trends in population abundance and distribution by gaining new knowledge on biodiversity is an essential step to achieve effective management and conservation measures at the gate of the Arctic, where anthropogenic activity is also expected to continue increasing in the near future^46,56^. Passive acoustic monitoring is the main way to gather data on marine soundscapes and provides new knowledge on the abundance and distribution of soniferous species as well as their surrounding environment. Our main goal was therefore to understand biotic and abiotic trends at the gateways to the Arctic and gain more insight into the seasonality of marine mammal acoustic diversity in these regions. Northern Norway provides an ideal example for an economically and ecologically important marine gateway to the Arctic, as it is likely to offer a wide range of sound types, complex oceanography, and anthropogenic activities. We examined the marine soundscape off the coast of LoVe over a period of five months to (1) quantify the variation in the biophony, anthrophony, and geophony in the local soundscape and (2) gain further knowledge on the temporal variation in occurrence of cetacean species. This is the first full soundscape analysis in coastal Norway and therefore serves as a useful foundation for monitoring species diversity and potential stressors at Arctic gateways, which will be valuable for future management initiatives.

## Results

**Figure 1:**
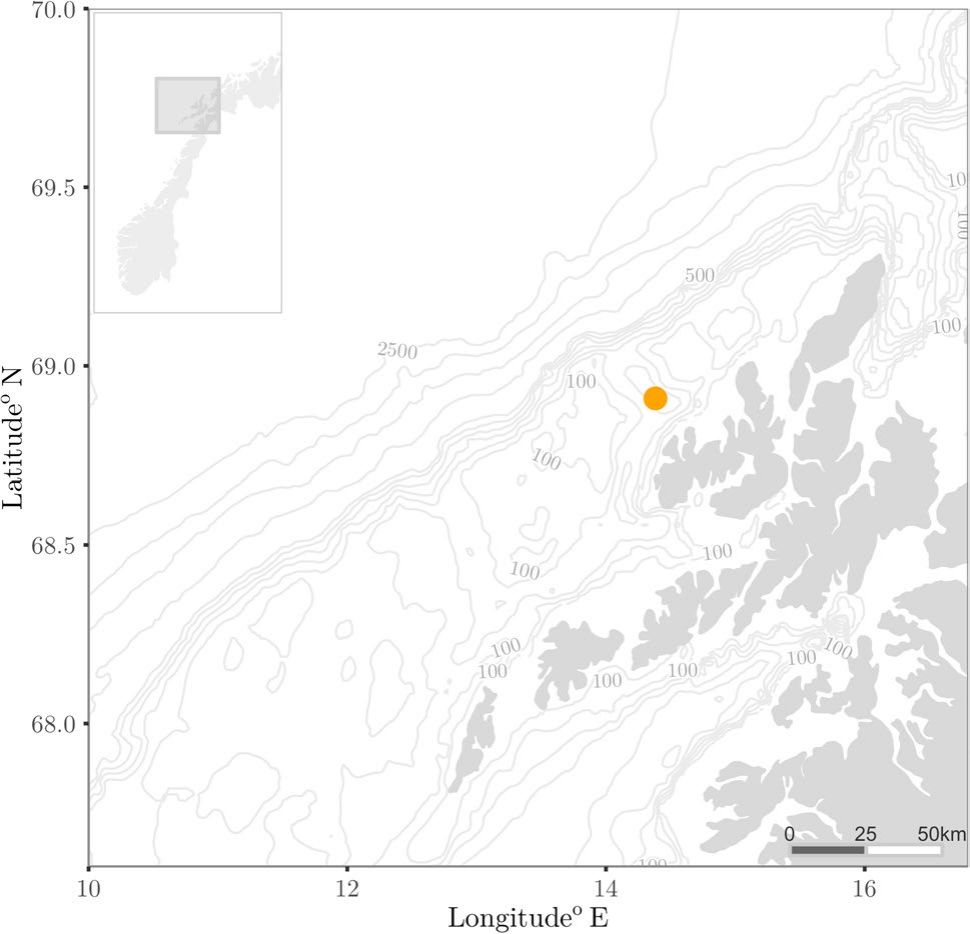
Map of the study area. The orange dot indicates the location of Node 1 of the LoVe Ocean Observatory. The figure was produced using the ggplot2^93^ and marmap^94^ R^87^ packages.

**Figure 2:**
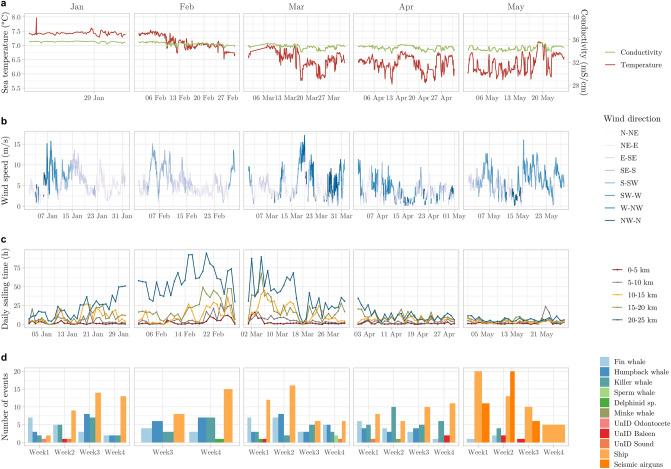
Overview of physical and acoustic properties of the sampling period. (**a**) Temperature and conductivity; (**b**) wind speed recorded by a land-based weather station; (**c**) cumulative sailing time across all ship types per distance radius; (**d**) detection events for each week of the analyzed months.

**Figure 3:**
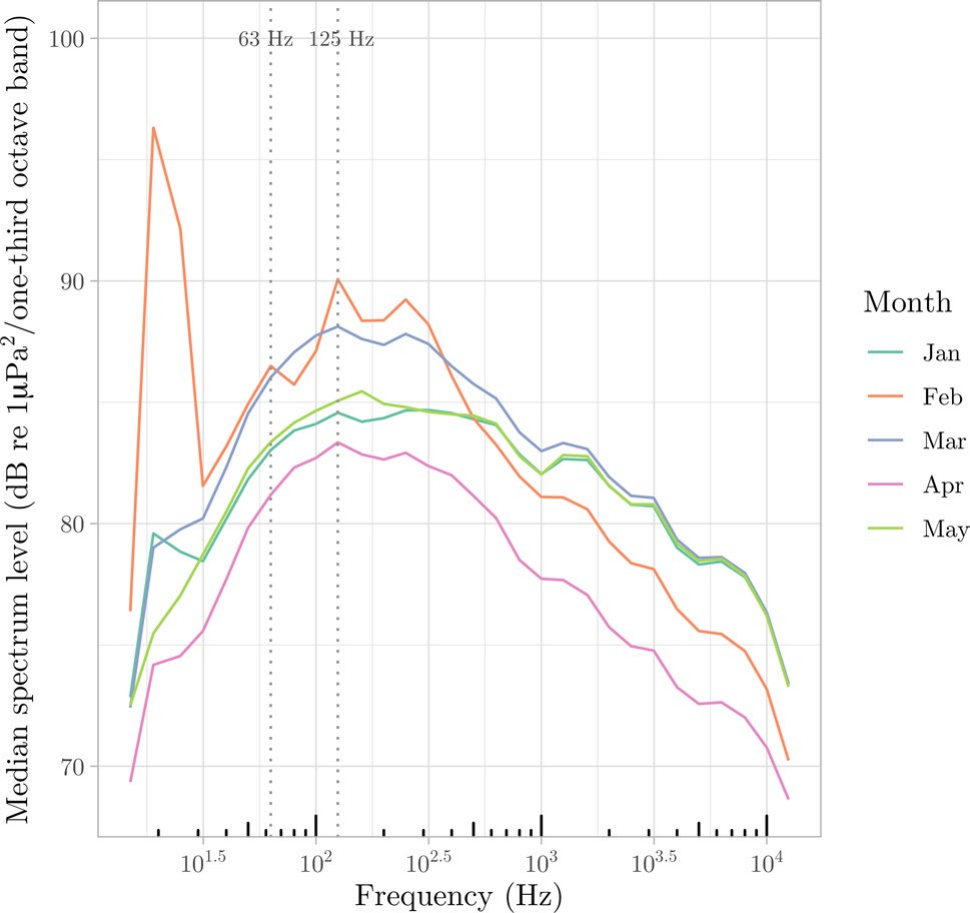
Median power Spectrum Density (PSD) curves for each month, calculated using 1/3 octave band measurements every 30 s. The vertical dotted lines indicate the 63 and 125 Hz centered 1/3 octave bands. Note that all values remain below the 100 dB threshold defined by Erbe (2013).

Marine sounds at the LoVe Ocean observatory (Fig. 1) had a very dynamic nature. Over the course of five months we sampled a total of 132 days of acoustic data. We report seasonal variations in physical features of the water column (temperature and salinity, Fig. 2a), wind speed (Fig. 2b), which is known to affect sound levels at low frequencies^57^, ship cumulative sailing time extracted from AIS data (Fig. 2c), and acoustic detection events of marine mammals and anthropogenic noise sources (Fig. 2d). Across the different months, median PSDs were highest in February and March (Fig. 3), ranging between 68.3 dB up 96.3 dB re 1 μPa^2^ (calculated at 1/3 octave bands) across all frequencies.

### Biophony

**Figure 4:**
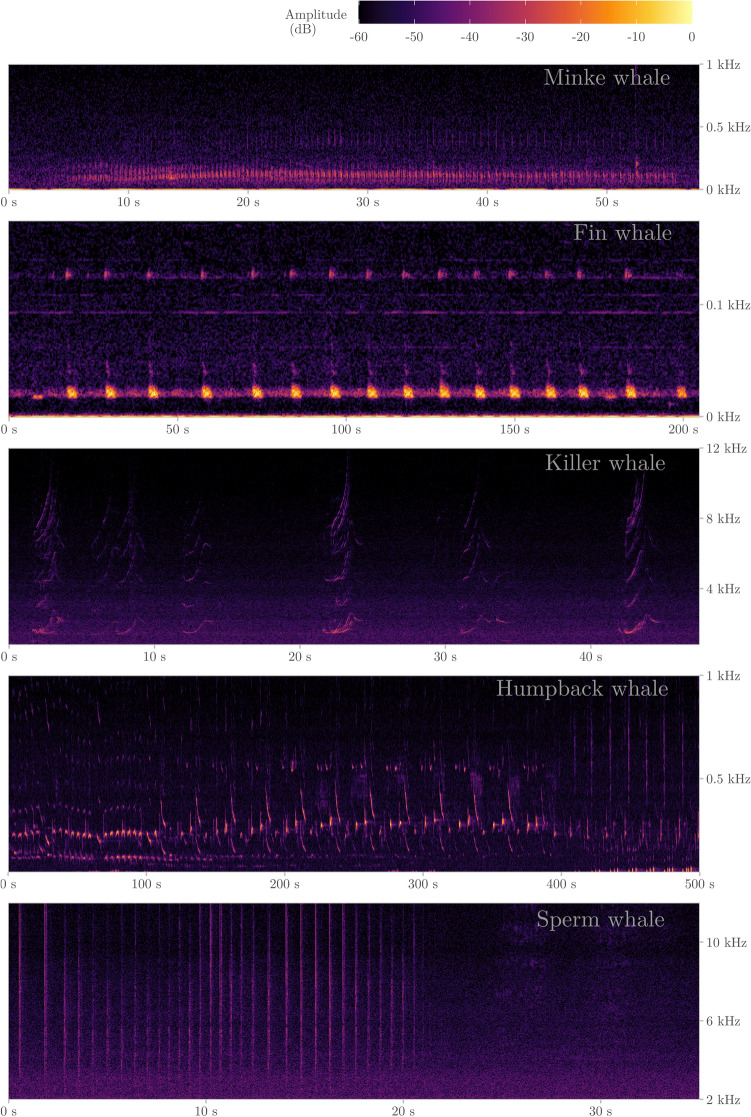
Spectrograms of five whale species detected in the acoustic dataset (all figures were obtained with Hanning window, fft size 4534 samples, and 50% overlap, with the exception of the fin whale which was done using an fft window size of 32,768 with 70% overlap, and the humpback whale with an fft window size of 8219 with 50% overlap).

**Figure 5:**
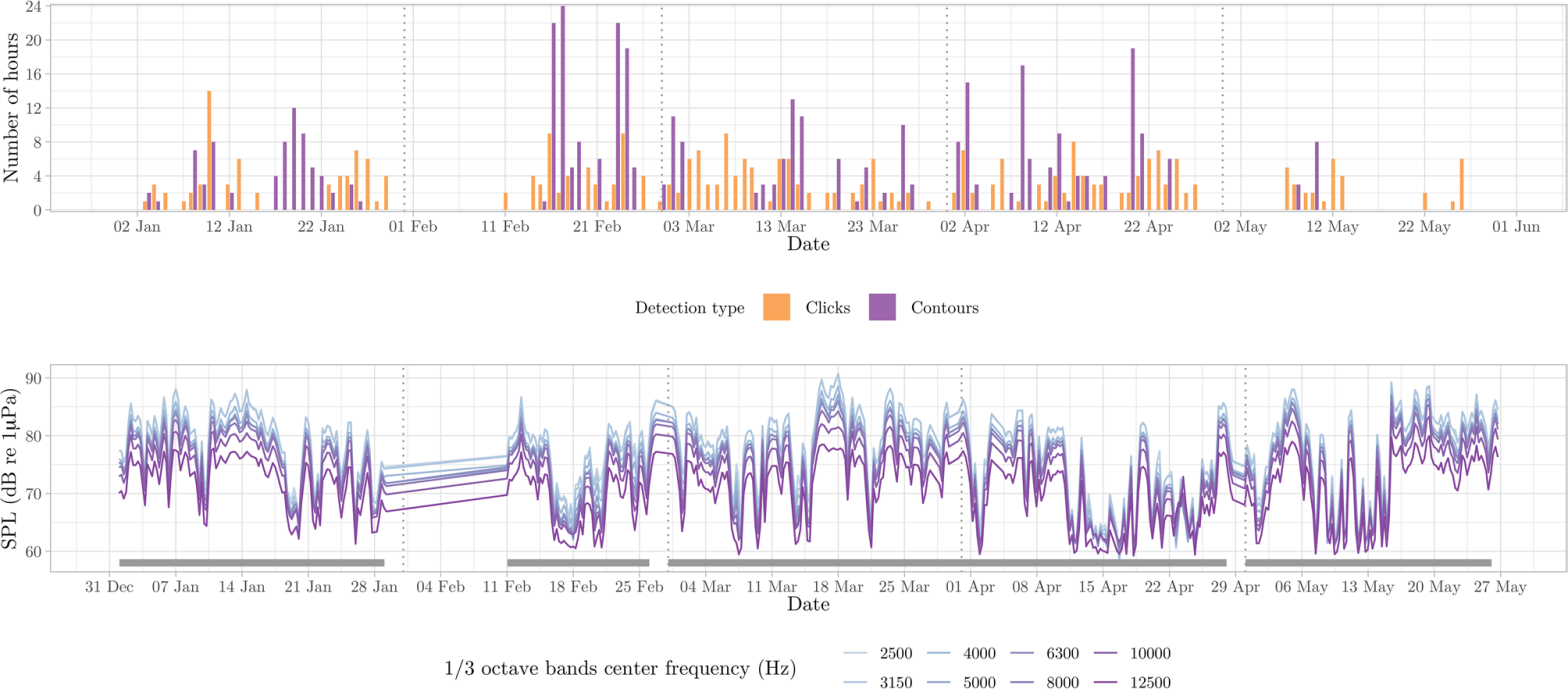
Number of detection hours per day for frequencies above 2 kHz, with detections according to the detector used for clicks, and contours, representing odontocete clicks and high frequency whistles (top). Sound Pressure Level values for different 1/3 octave bands, grouped into six-hour averages for visibility purposes (bottom). Grey segment bars indicate the sampling effort in days (i.e., gaps represent lack of acoustic data).

**Figure 6:**
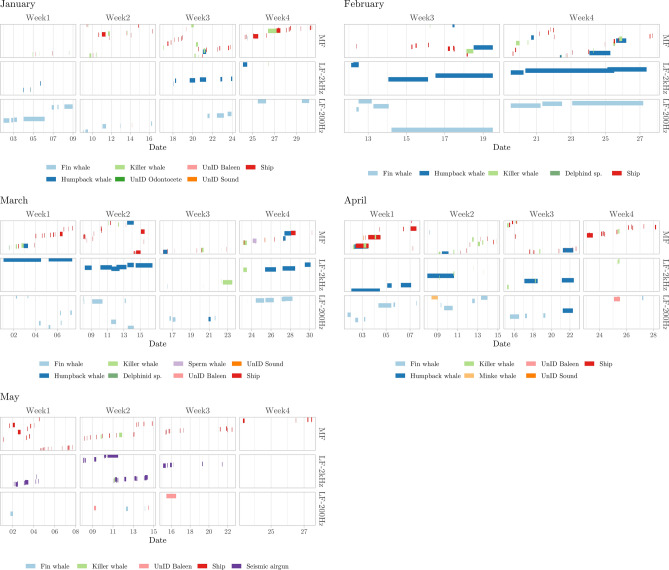
Overview of detection events for each week of each month and detection band (LF-200 Hz refers to frequencies between 0 and 200 Hz, LF-2 kHz refers to frequencies between 200 Hz and 2 kHz, and MF refers to frequencies above 2 kHz). The y axis represents the order in which the data were processed. Segment length indicates event duration.

**Figure 7:**
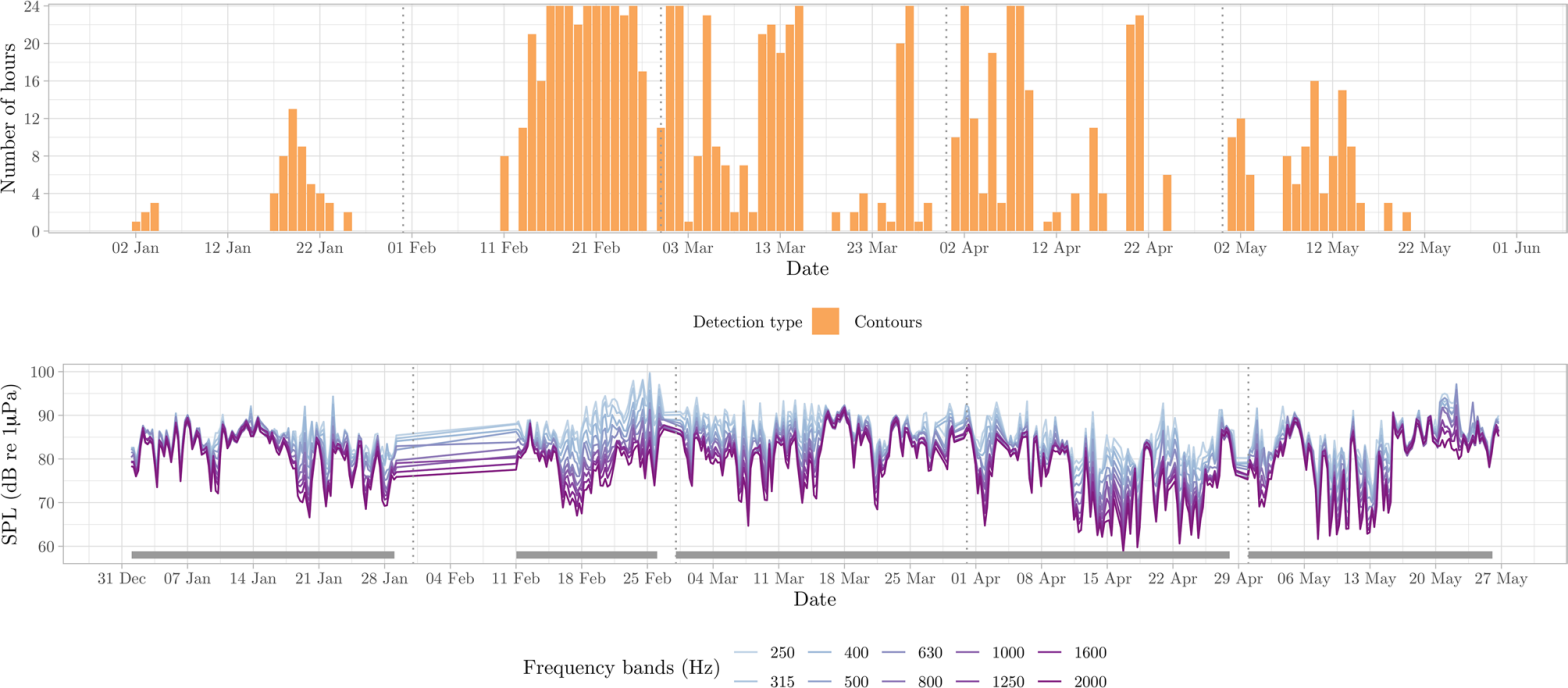
Number of detection hours per day for frequencies between 200 Hz and 2 kHz, with contour detections representing modulated signals of humpback whales and odontocetes (top). Sound Pressure Level values for different 1/3 octave bands, grouped into 6-hour averages for visibility purposes (bottom). Grey segment bars indicate the sampling effort in days (i.e., gaps represent lack of acoustic data).

**Figure 8:**
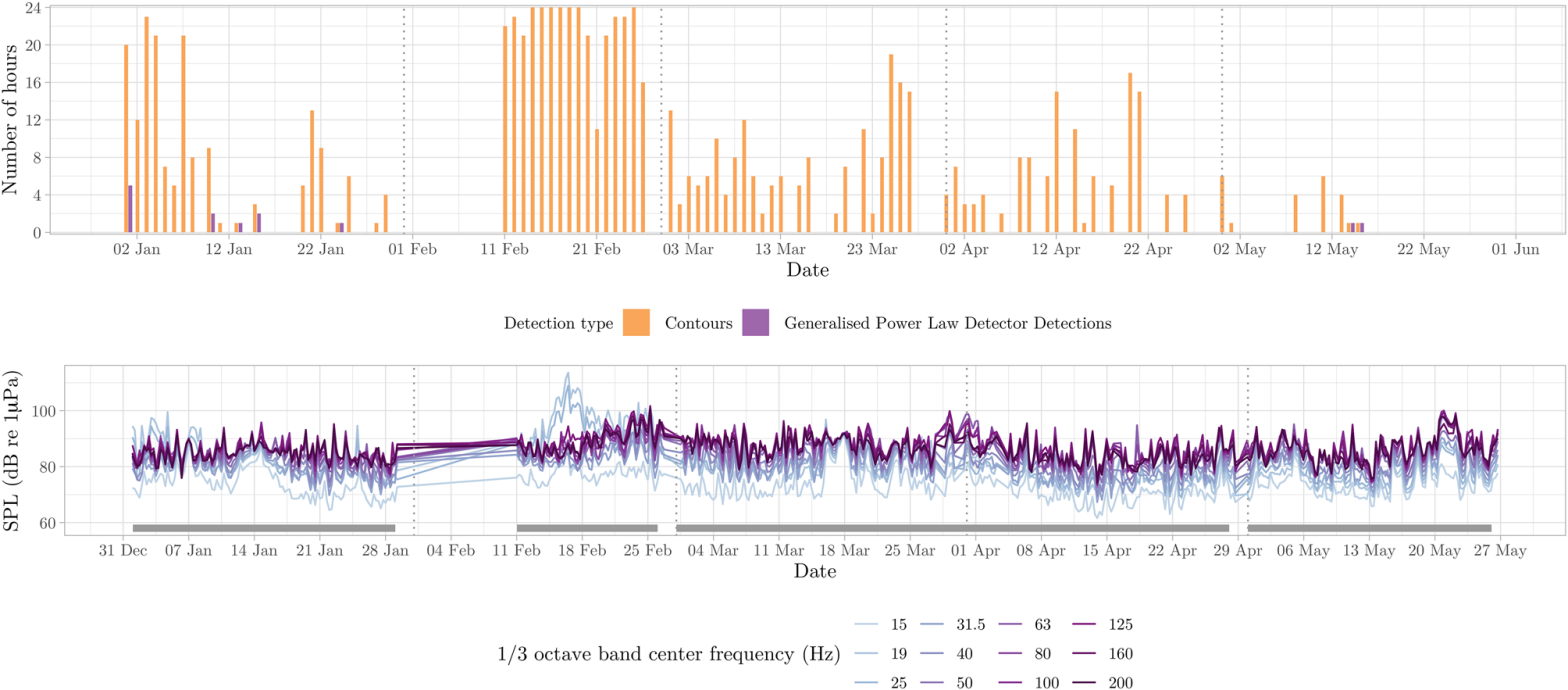
Number of detection hours per day for low frequency detections between 0 and 200 Hz, with detections for contours and generalized power law, representing low-frequency modulated signals generally associated to baleen whale species (top). Sound Pressure Level values for different 1/3 octave bands, grouped into six-hour averages for visibility purposes (bottom). Grey segment bars indicate the sampling effort in days (i.e., gaps represent lack of acoustic data).

**Figure 9:**
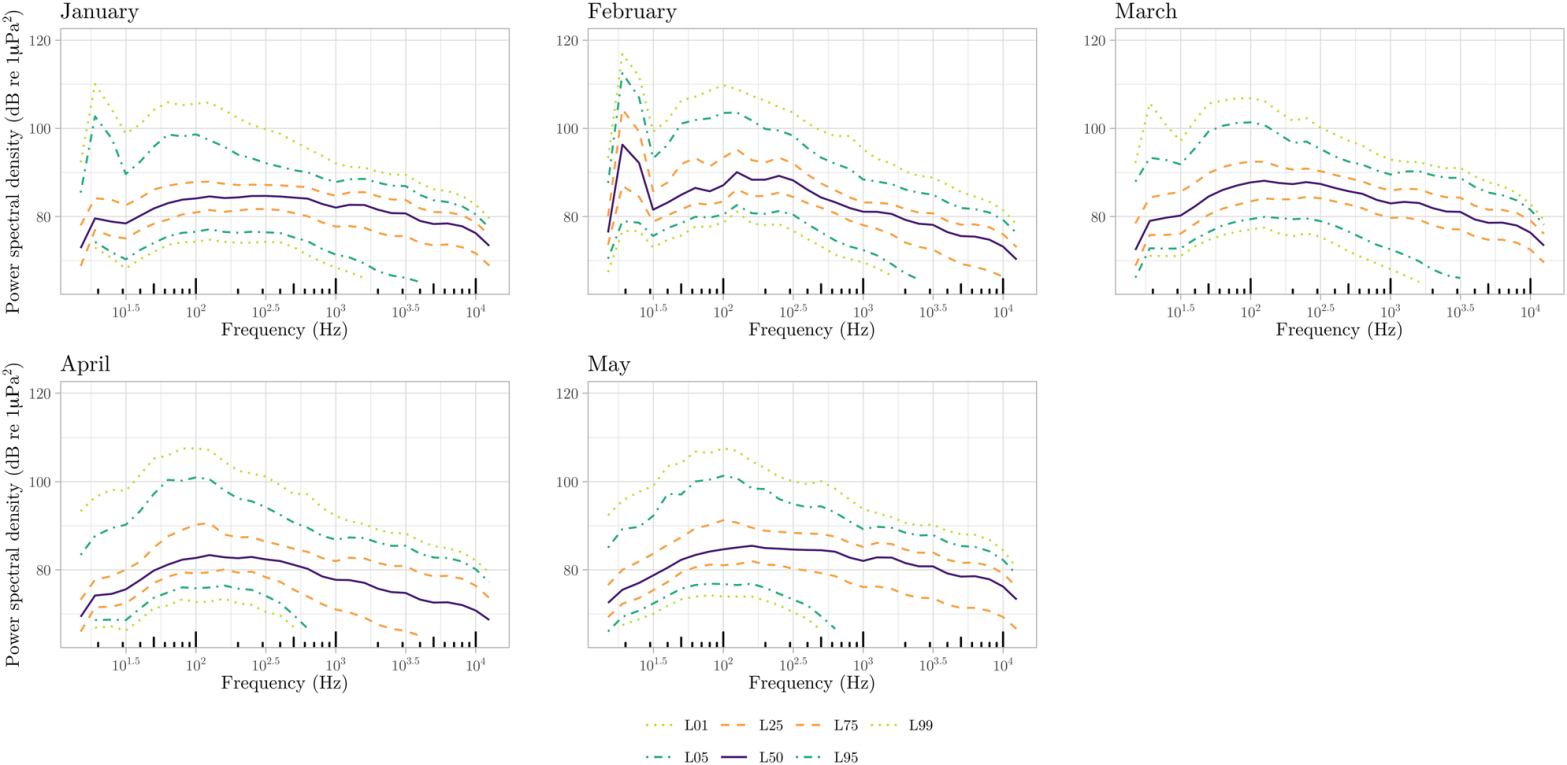
PSD curves with probability distribution percentiles (L01—1%, L05—5%, L25—25%, L50—50% also known as median, L75—75%, L95—95%, and L99—99%) for each month.

We identified calls from five different cetacean species in the LoVe recordings (Figs. 2d and 4). The seasonality of the detected calls likely represents changes in distribution related to the species’ migratory behavior and foraging activity. March was the month with most biological events detected (Fig. 2, Table 1). Vocalizations at frequencies above 2 kHz were dominated by clicks and modulated calls (Fig. 5), mainly corresponding to humpback whale harmonics and delphinids (Fig. 6). Killer whales were present in all sampling months, with a peak in the number of events in April (Table 1). Though only a few events were detected, sperm whales and minke whales appeared occasionally during the spring-onset (Figs. 2d and 6; Table 1). Humpback whales were engaged in singing from mid-January to the end of March, dominating frequencies up to 2 kHz. The longest detection event duration belonged to humpback and fin whales (Fig. 6), coinciding with peaks in the number of hours with vocalizations, particularly in the end of February up to 2 kHz (Figs. 7 and 8). Fin whale 20-Hz calls occurred from early January to late April, and also visible in peaks of varied amplitudes in PSD plots (Fig. 9).

**Table 1:** Number of events for each detection group (whale species and anthropogenic sources) per month. UnID indicates animal groups unidentified to the species level.

	Jan	Feb	Mar	Apr	May
Fin whale	17	7	22	14	3
Humpback whale	13	13	19	11	0
Killer whale	16	10	13	26	6
Sperm whale	0	0	2	0	0
Minke whale	0	0	0	1	0
Delphinid	0	1	1	0	0
UnID odontocete	1	0	0	0	0
UnID baleen	1	0	1	2	3
UnID sound	1	0	1	1	0
Ships	38	23	40	35	54
Airguns	0	0	0	0	37
Total	87	54	99	90	103

### Anthrophony

**Figure 10:**
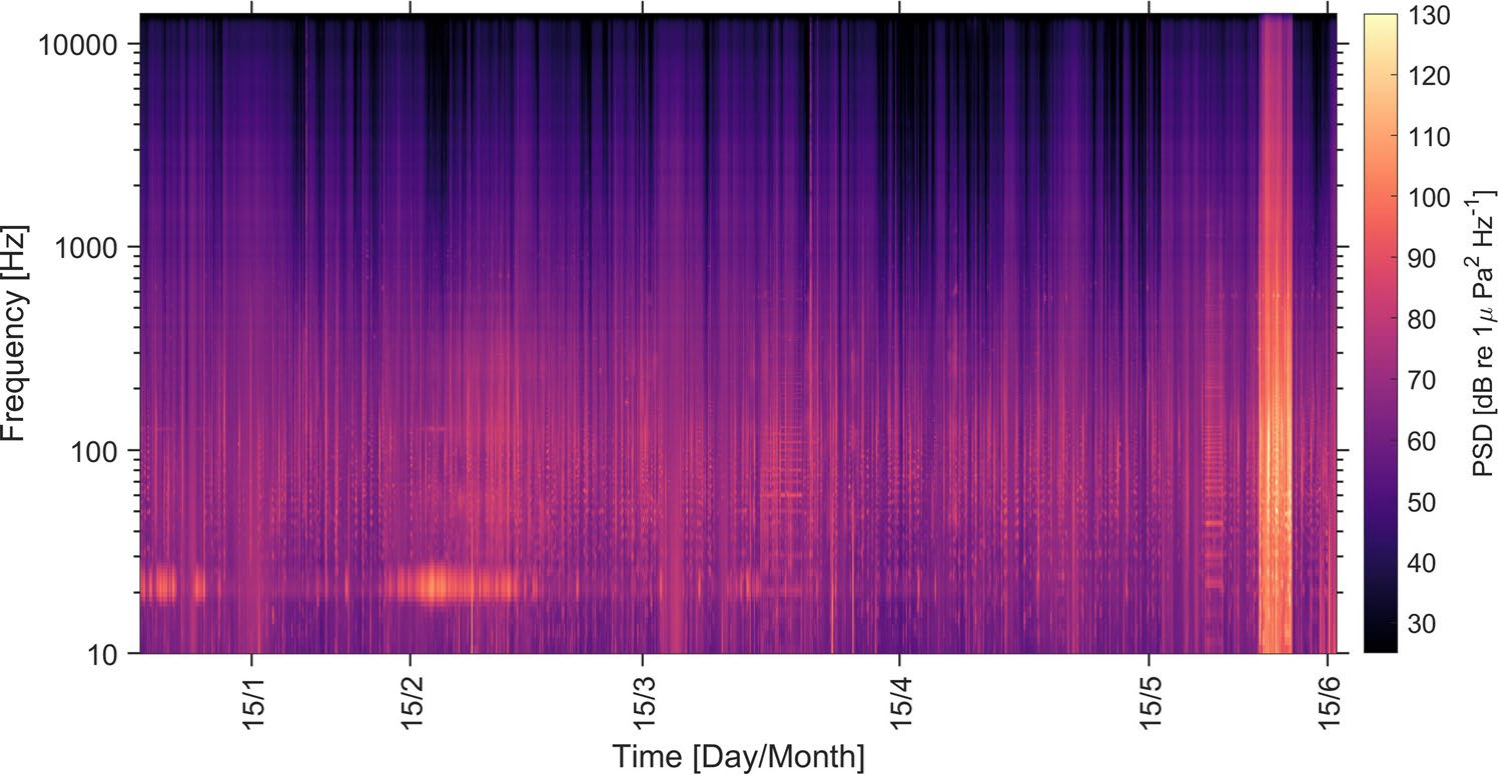
Long-term spectral average (LTSA) plot for the study period, calculated in Matlab following Merchant (2015) with a frequency resolution of 1 Hz and time-averaged over 10 s using the Welch method. Frequency is displayed in log form on the y-axis.

The influence of ship traffic, as a component of the local anthrophony, was clearly distinguishable from biological sounds in the LTSA (Long-Term Spectral Average) spectrograms (e.g. Fig. 10). During the review of the audio files in PAMguard, we recorded ship detection events throughout the study period. From the events, we can see that the number of vessel passages recorded by the hydrophone was fairly consistent throughout the months of January to April (Table 1). May was the month with most vessel events detected (Table 1). Airgun surveys were detected solely in May (Table 1). From the daily estimates of SPL for the different 1/3 octave bands, we observed a significant difference between median spectrum levels of the two main frequency bands associated with low frequency anthropogenic sounds (centered at 63 and 125 Hz, Wilcoxon test p = 8.5 × 10^–5^). This difference is also visible in the power spectrum estimates for the different months, where February had highest activity and April lowest (Fig. 7).

### Geophony

Through inspection of long-term spectral averages (Fig. 7), we observed periods of occurrence of breaking waves (e.g., around the 15th of January and mid-March). Particularly during winter and spring, these events seem more pronounced, coinciding with a tendency for higher occurrence of storms and strong wind velocities during the same period of the year. Because of the large density of vessel passages and cetacean occurrence across frequency bands often associated with weather (e.g., > 1 kHz considering bubbles and spray related to surface agitation^57^), we have not considered results concerning the seasonal variation of weather-related sounds.

### Effects of environmental and human activity on sound levels

The model structure selected was similar across the three frequency bands tested. The results of the models selected are summarized in Table 2. When testing the effect of ship traffic across cumulative distance ranges for all vessel types (that is, 0–5, 0–10, 0–15, 0–20, and 0–25 km), the model corresponding to sailing time within 10 km had the most support from our data for frequencies below 200 Hz. For frequencies above 200 Hz however, the most supported model was defined by vessels within the 5 km range (that is, for frequencies in the range of 200 Hz to 2 kHz and above 2 kHz). Given that, for frequencies below 200 Hz, the best models were represented by vessels within the 10 km range, we made a further model selection step to assess differences in model fit between singular bins (that is, 0–5 km, 5–10 km). Though the model for the 0–10 km range provided a lower AIC value, the distribution of the residuals was not considered satisfactory in relation to the distribution provided by the models for the subsequent bins. We found that between the two models (0–5 km and 5–10 km), the model with the best fit was represented by all vessels in the 5–10 km range. Additionally, we detected an outlier in SPL measurements, representing a day with exceptional mean SPL values (100 dB re 1 μPa). This outlier did not appear to cause any change in relationship trends between predictor and response variables nor model selection results, and was therefore removed from further data examination. Upon discrimination of vessel type per selected distance bin, we discovered a significant effect in SPL values at frequencies below 200 Hz for vessels of the “fishing”, “cargo”, “tanker”, and “other” categories. For frequencies above 200 Hz (0–5 km range), we found a significant effect of passenger and cargo vessels. Wind speed was found significant in all models. Results from the model selection process, with discriminating estimates for each tested model, respective AIC scores, and diagnostic plots, are presented in S2 and S3.

**Table 2:** Statistical model results for the three models selected.

	< 200 Hz			200 to 2000 Hz			> 2000 Hz		
Coeffcient	Estimates	Conf. int (95%)	P-value	Estimates	Conf. int (95%)	P-value	Estimates	Conf. int (95%)	P-value
Intercept	83.24	81.89–84.59	** < 0.001**	82.20	81.06–83.34	** < 0.001**	75.14	73.81–76.46	** < 0.001**
Wind speed	1.04	0.66–1.42	** < 0.001**	2.50	1.99–3.01	** < 0.001**	3.53	2.83–4.23	** < 0.001**
Sailing time 5–10 km fishing	0.68	0.34–1.01	** < 0.001**						
Sailing time 5–10 km passenger	− 0.03	− 0.33–0.27	0.855						
Sailing time 5–10 km cargo	0.89	0.64–1.14	** < 0.001**						
Sailing time 5–10 km tanker	0.31	0.06–0.56	**0.016**						
Sailing time 5–10 km other	0.85	0.40–1.30	** < 0.001**						
Sailing time 0–5 km fishing				0.20	− 0.19–0.59	0.314	− 0.40	− 0.95–0.15	0.155
Sailing time 0–5 km passenger				0.39	0.03–0.75	**0.034**	0.46	− 0.05–0.97	0.076
Sailing time 0–5 km cargo				0.78	0.44–1.13	** < 0.001**	0.62	0.13–1.11	**0.013**
Sailing time 0–5 km tanker				− 0.09	− 0.46–0.28	0.625	-0.10	− 0.62–0.42	0.706
Sailing time 0–5 km other				0.14	− 0.35–0.62	0.574	-0.21	− 0.88–0.47	0.544
Observations	132			132			132		
R^2^	0.350			0.500			0.509		

Significant values are in bold.

## Discussion and conclusions

The current knowledge gaps on multi-seasonal occurrence of top marine mammals in Norway and the increased climatic and ecological pressures at the Arctic borders make the LoVe region a good example to showcase acoustic variation occurring at the gates of the high-Arctic. Our work provides an understanding of interactions between biophony, anthrophony, and geophony while establishing a cross-season baseline for future work in measuring the level of acoustic variation in years ahead. Our results also inform changes in biological diversity during part of the polar night and spring which has so far been undocumented. Furthermore, we show the potential of conducting acoustic research and monitoring in areas and periods with challenging operational conditions. Unlike traditional surveys of marine biodiversity, which are potentially affected by human presence, acoustic records can be autonomously collected, twenty-four hours a day, over long times and also under adverse meteorological conditions^20^.

### Patterns in biophony

Marine mammals are a vital component of oceanic soundscapes, and as top predators of marine ecosystems, monitoring their seasonal presence^58^, long-term temporal trends^33,34,59^, migration patterns^6,60^, behavior^10,61,62^, distribution^6,63^, and abundance^64,65^, ultimately provide an understanding on ecosystem health. Several studies have highlighted the value of passive acoustic monitoring (PAM) for marine mammal monitoring and research^18,24,66^. Particularly in periods of low visibility or areas of difficult access, this method can provide insights into the behavior of cetaceans in a non-invasive matter. Our findings show that the LoVe area represents an important location for whale communication with dynamic species occurrence across multiple seasons. Furthermore, we observed a large diversity of species across all months sampled, highlighting the importance of monitoring across multiple seasons. Ultimately, our detection of marine mammals not only represents an increase in local biodiversity, but also reflects ecological conditions suitable for cetacean foraging and social exchange. Events of biological activity were dominant during early spring and consisted of a combination cetacean species and unidentified biological sounds. We observed notable acoustic signals by humpback and fin whales throughout winter and early spring. Among the calls detected was the humpback whale song, which occurred predominantly from late January to mid-April. Fin whales are known to occur mainly in the summer though some off-season sightings are not exceptional^67^. Fin whale calls were persistent throughout the study period and detected systematically every week of data analyzed. Humpback whales have been reported from October–April in the Norwegian Sea in connection to the distribution of the Norwegian spring-spawning herring (*Clupea harengus*)^38,40,68^. Though our results provide further insight into their seasonal occurrence, we also show the value of the LoVe region for cultural transmission for this species, and a first glimpse of the co-occurrence of humpback whale song and anthropogenic noise. Odontocete calls occurred in all sampled months, though the number of sperm whale detections were too low to gain any insight onto their site use. Yet, male sperm whales are known to forage in the LoVe region throughout the year^40,43,69^. Pedersen et al. reported seasonal mesopelagic layers containing fish and squid, with simultaneous occurrence of sperm whale foraging clicks at the LoVe Ocean observatory. Similarly, killer whales tend to move in the Norwegian Sea in function of prey distribution^70^ and may participate in multi-species aggregations when foraging for common prey^41^. It is therefore likely that the seasonal occurrence of mesopelagic prey is a decisive factor in killer whale distribution, and therefore detection by the PAM recording system.

### Patterns in anthrophony

Underwater sounds generated by human activity are often connected to the shipping industry^71^. Accordingly, vessel passages were detected every week of available data from LoVe Ocean. The recorded sounds attributed to vessel passages have likely two main sources: a shipping lane 55 km northwest of the recording station, and local vessel traffic. The occurrence of sound channels could indeed aid in the propagation of low frequency signals originating from the shipping lane, though the present study did not conduct acoustic modelling to verify whether this is the case. In any case, increases in sound levels are more likely due to increases in local vessel traffic. Though shipping noise is generally associated to frequencies below 1000 Hz^57^, the frequencies at which these noise sources dominate depend on the type of vessel, load, and speed^72^. Anthropogenic events were rather consistent over the sampling period, with a peak in May. These events were mainly represented by vessels operating in the region. We detected a fairly consistent number of ship passage events from Winter to late Spring, which is not visible in the the sailing time estimated from AIS data. However, this could be due to trade-off between variations in the acoustic background, which can affect the the acoustic detection of vessel passages versus their duration and the information provided by the AIS system. Upon assessing levels of underwater sound in relation to sailing time, our models show seasonal patterns.

For the two main frequency bands associated with long range shipping signals (centered at 63 and 125 Hz 1/3 octave bands) by the European Union’s Marine Strategy Framework Directive^73^, we observed that February was the month with highest noise levels. Though guidelines have been established to aim for levels below 100 dB^74^, increases in sound levels at those two bands may not necessarily be connected to vessel traffic^75,76^ and demand further scrutiny. Nevertheless, all median spectrum levels remained below this threshold.

We observed an increase in estimated SPL values under 200 Hz with increasing sailing time for fishing vessels, cargo, tanker, and “other” ship types within 10 km from the recording station. Passenger vessels were the only ones to not show any statistical significance at that distance range and frequency values. At frequencies higher than 200 Hz, we found that cumulative values of passenger and cargo vessels have a positive effect within 5 km from the observatory. While both were significant for frequency ranges between 200 Hz and 2 kHz, only cargo ships were found to be significant for frequencies above 2 kHz. This indicates that the contribution of cargo vessels to the overall soundscape surpasses other vessel activity. Though Lofoten–Vesterålen is a notorious region for its fishing activity, coastal vessel traffic is largely represented by cargo vessels, which is visible in our results. Yet, another possible reason for these findings is the conditions for high frequency-sound propagation in the region and the bathymetric location of the recording station in the small underwater canyon. Nevertheless, we were able to document the effect of vessels at distances up to 10 km onto SPL values on frequencies below 200 Hz, highlighting the levels of acoustic pollution driven by human activities in the area.

### Patterns in geophony

Previous studies have investigated the effect of geophony and low frequency sounds at the LoVe Ocean observatory. In 2014, Ødegaard et al.^56^ found that wind-generated noise at low-frequencies (< 400 Hz) is higher at the observatory than reported in Wenz curves^57,77^. There is also evidence of occurrence of long-range propagating earthquakes for the 2018 dataset^72^, coinciding with peaks in our spectrum density results.

We found that wind speed had a significant effect on SPL values across all frequency bands tested. However, the total variance explained by our model suggests that there may be confounding factors that were unaccounted for in this study. Though we acknowledge that wind speed and vessel type within the selected bins alone are not enough to best describe received sound levels, it was not possible at this stage to consider complementary measures (e.g., currents and swells) that could also affect them.

Overall, our results highlight the potential for using the combination of AIS data/recorder ship detections, meteorological sampling, and marine mammal calls to evaluate the effects of noise on marine mammals and assess seasonality in future studies. Still, according to the Norwegian Costal Administration fishing vessels smaller than 15 m in length, and all other vessels with less than 300 gross tonnage, are not required to use AIS, and those that do meet reporting requirements may also turn off the AIS system. Ship noise source level can be different with varying speed through water, and stormy weather could result in higher ambient noise levels^57^. Furthermore, the travel direction of the vessels may also have an impact on received levels^78^. An assessment of these interactions is therefore advisable in future soundscape endeavours. To this point, the LoVe Ocean region still seems to be affected by long-range shipping noise derived from fishing vessels and close range large tonnage vessels such as cargo and passenger ships. Another issue to highlight is the detection of airgun pulses which, despite the ban on oil prospecting in the region, were detected throughout late spring. This raises the need for considerations on the effects of long-range acoustic propagating activities, which may disrupt wildlife in areas categorized as protected.

Underwater noise and its effects on marine biodiversity has been receiving increasing attention, with recognition by international and regional agencies, commissions, and organizations. Though such initiatives are of great value to mitigate the effects of underwater noise resulting from anthropogenic activity, it is imperative to also consider the preservation of ecosystem structure and function by ensuring the conservation of natural soundscapes. In face of climatic change and expansion of anthropogenic activities into northern regions, it is increasingly important to investigate seasonal variations across components of the soundscape. Most of the biological events detected in this study belonged to marine mammals and encompassed a diverse range of vocalizations that may provide indications onto their foraging and social behavior. More detailed assessments on annual variability of species occurrence and their behavior would therefore be merited to address the acoustic component of phenological change. Furthermore, monitoring marine sound to investigate the occurrence of sudden and extreme climatic events can provide insights onto effects for acoustic detection and implications to biodiversity.

Based on the current status of the LoVe soundscape, we propose systematic acoustic monitoring to be included in future management plans in areas of transition into the Arctic. At the moment, the LoVe Ocean Observatory is the only station recording soundscapes between Northern Norway and the Barents Sea. Studies including the summer months, large arrays of recording systems, and across multiple years would be valuable to add on to the information provided in this study and allow for more knowledge on acoustic diversity, spatial distribution, and annual animal occurrence.

## Methods

### Survey area and acoustic recording

We analyzed passive acoustic data collected over a period of 6 months from a cabled ocean observatory (LoVe Ocean, located at N 68° 54′, E 14° 23′). This seabed structure was first deployed in 2013 in a sub-sea valley about 20 km off the coast of Vesterålen, Norway^51^. The observatory lies in the Norwegian Sea, in the vicinity of the Lofoten archipelago, where the continental shelf is relatively shallow^79^. With the intention of collecting a wide range of biological sounds, the recording station (first node of the observatory) was located at the entrance of a natural shallow canyon, the Hola valley at a depth of 258 m (Fig. 9). Other nodes have been implemented with additional sensors though in this analysis we present singly hydrophone data from Node 1^77^. We present data collected from January 2nd to May 28th 2018, using a SB35 ETH hydrophone (Ocean Sonics) with a bandwidth of 13 Hz–16 kHz and response sensitivity of – 171 dBV re. 1 μPa, assuming a stable frequency response, and Zero-to-peak voltage 3.0 Vpk. The hydrophone was set to record continuously in 10-min segments, though there were gaps in acoustic recordings owing to maintenance duties and power outages (see Figs. 2, 3, and 4 for survey effort).

### Acoustic processing

All passive acoustic data were processed using semi-automated methods in PAMGuard (version 2.01.05)^80^. In order to investigate temporal trends across the entire sound frequency spectrum, we generated long-term spectral averages (LTSAs) for all files and displaying scaled colors for amplitude values present in the recordings. Additionally, we calculated average 1/3 octave-band pressure levels (SPLs, dB re 1 μPa, rms**)** every 30 s for all 10-min files (50% overlap, Hanning window). The 1/3 octave band analyses were performed within the framework of Descriptor 11 or the European Marine Strategy Framework Directive 2008/56/EC11 for marine noise monitoring^73^. In total, we calculated average SPL values for 33 1/3 octaves starting at 13–17 Hz and up to 14,254–17,959 Hz, including the designated bands for long range propagation centered at 63 and 125 Hz in European waters^73^. Each 1/3 octave SPL was then averaged in 6-h bins and used to produce a visual representation of the variation over the entire sampling period. We further assessed daily estimates in median spectrum levels for the two 1/3 octave bands corresponding to long-range propation (centered at 63 and 125 Hz) using a Wilcoxon test^81^.

### Biophony

We designed three configurations which incorporated customization of the inbuilt PAMGuard detectors (the “Whistle and Moan” for all tonal and burst pulsed calls, the “Generalized Power Law” detector for baleen whales, and the parameterized “Click” detector for echolocating species) in order to estimate call occurrence. Each configuration was designed to analyze a frequency band; 10 to 200 Hz, 200 Hz to 2000 Hz, and 2 kHz to 12 kHz. This allowed for an improved discrimination method between species, while permitting more specificity when looking for animal groups naturally occurring in the region.

Though killer whales and sperm whales are the two main resident species in coastal Norway^43,82^, few reports document humpback and killer whales winter foraging aggregations in the region^38,41^. Fin whales, on the other hand, have only been found acoustically in the area during autumn and winter^54^. Data for the lowest frequency processing (10–200 Hz) were decimated to 400 Hz sampling rate to improve detector performance. All automated detections were logged in PAMGuard’s Viewer Mode, assigned to a detection group, and classified as detection events. A detection was classified as a separate event when the time between vocalizations was longer than 30 min for odontocentes species and 60 min for baleen whale species. This was done to account for a diversity in vocalization rates and diving bouts across locally occurring species. Cetacean species were manually identified by an experienced acoustician through aural and visual inspection of spectrograms for all automated detections (FFT_< 0.2 kHz_ = 256; FFT_0.2–2 kHz_ = 1024; FFT_> 2 kHz_ = 1024; 50% overlap and Hanning window across these ranges). A species or species group was only assigned if the characteristics of calls matched those of well documented calls produced by the same species within the literature for this ocean basin^83–85^. For detections when this was not possible, we classified them to sub-Order (Baleen or Odontocete) or Unidentified biological sound. Duplicate calls belonging to the same acoustic event but detected across multiple configurations were removed based on the timestamp of the detection.

### Anthrophony

As part of the event classification mentioned above, we classified recorded ship sounds by reviewing LTSA plots and aurally analyzing to ensure non-biological activity. We collected information on ship traffic by examining ship traffic data for vessels within 25 km from the sampling station, subsampled to represent vessels in at open sea (i.e., not in fjords nearby). Ship traffic data were provided by the Norwegian Coastal Administration and consisted in a tracking dataset based on the Automatic Identification System (AIS) developed by the International Maritime Organization^86^. The AIS tracking is currently mandatory for fishing vessels above 15 m in length and for other vessels above 300 gross tonnage. Smaller vessels are not part of this dataset and were thus not included in our analyses. AIS data processing was done using R Statistical Software v4.0.3^87^. The AIS data were first filtered for anomalies (positions on land or generating unrealistic travel speeds, wrong identification numbers). We then generated a georeferenced grid at 0.01 × 0.01° spatial resolution before computing the daily cumulative sailing time of all ships in each pixel, using function tripGrid from package trip v.1.8.5^88,89^. Cumulative sailing time was defined as the amount of time (hours per day) spent by vessels within different ranges from the hydrophone (0–5 km, 5–10 km, 10–15 km, 15–20 km, and 20–25 km) (S1). Ship traffic data from zones that were not in direct sight of the hydrophone (e.g., hidden by large islands) were excluded (Fig. S1). SPL measures associated with ship traffic were aggregated into daily averages to match the temporal resolution of vessel sailing time.

### Geophony

Wind speed was considered the principal factor that influences the sea state condition and, consequently, the noise generated by breaking waves^17^. We obtained time series data from the nearest weather station located on land (in Bø) as a measure of current wind conditions in the area. Though we acknowledge the complexity of sound propagation and sources that may affect the resulting SPL values across multiple frequencies, it is beyond the scope of this study to investigate the effects of current direction, swell, and topography on the sampled geophony.

### Effects of human and weather activity on recorded sound levels

Determining seasonal fluctuations and periods of higher or lower correlation is of relevance to determine fine scale variations of the effects of predictor variables on received SPL values. Using R^87^ software, we investigated the acoustic environment animals may be exposed to, by analyzing the resulting time series for daily SPL values, sailing time (anthrophony), and wind (geophony). Given our classification of whale encounters as weekly events and the considerable large proportion of whale presence on a daily basis, we did not consider animal vocalizations as valuable contributor to the overall soundscape^90^. Considering the effects of anthropogenic activity and contributions of biological sounds to the soundscape, we assessed the statistical effect of daily sailing time per vessel type for each distance range, wind speed, and wind direction (as a categorical variable) on the daily averaged SPL values for three frequency bins (< 200 Hz, 200–2000 Hz, and > 2000 Hz). We chose to use these bins for two reasons: (1) we can relate findings to biological detections in similar bins, and (2) to ensure that we account for possible relationships beyond expected frequencies. We selected the daily resolution given that the analyses rely on vessel density rather than raw AIS information. From the AIS data, we acquired information on sailing time for five vessel categories: fishing, passenger, cargo, tanker, and other. Vessels under the “other” category were included to combine ships that do not belong to any of the other categories, and could include vessels such as supply ships, rescue vessels, or coastguard. Because AIS data in distance bins are naturally autocorrelated (i.e., the same vessels can cross different bins), we tested generalized least squares models that included sailing time at each individual distance range, cumulative ranges, and vessel type separately using the gls function (nlme package^91^). We included wind speed, and vessel sailing time per distance range as fixed parameters, and an autocorrelation term with a continuous autoregressive structure for Julian Date to account for temporal correlation in ship traffic data among successive days. We tested models that also included an interaction term to account for possible dependencies between wind speed (numeric variable) and direction (4-level categorical variable). This interaction was found not to be significant for our location and was subsequently removed from the further model selection process. Model selection was made by comparing the Akaike Information Criterion (AIC) using the aictab function of the AICcmodavg package^92^.

Finally, graphics for event distribution, time series, PSD plots, and map were produced using the ggplot2^93^ and marmap^94^ packages, with high resolution geography data from the GSHHG (Global Self-consistent, Hierarchical, High resolution Geography) database^95^. Spectrograms were produced using an adapted version of the ggspectro function of the seewave package^96^. LTSA visualizations were produced in Matlab^97^ and PAMGuide^98^ software.

## Supplementary Information

Supplementary Information.

## Data Availability

The audio files generated during the current study are available from love.equinor.no and loveocean.no. All other datasets, with the exception of raw (i.e. GPS tracks of individual ships) AIS data, are available upon reasonable request to the corresponding author.
