# Supplementary material for: Temporal patterns in the soundscape of a Norwegian gateway to the Arctic
Source: Sci Rep. 2022 May 10;12:7655. doi: 10.1038/s41598-022-11183-y (PMC9090731; doi:10.1038/s41598-022-11183-y)
Supplement: Supplementary file 1 — Supplementary Information. [file 41598_2022_11183_MOESM1_ESM.docx]

# Supplementary information

S1: Map of the sampling area for the vessel sailing time dataset. The marked cross represents the location of the acoustic recording station of the LoVe Ocean Observatory, with the distance radii used in estimates of effects of sailing time on sound levels (0-5 km, 5-10 km, 10-15 km, 15-20 km, 20 -25 km). The figure was made using the ggplot ^93^ R ^87^ package. High resolution geography data was acquired through the GSHHG (Global Self-consistent, Hierarchical, High-resolution Geography) database ^98^.


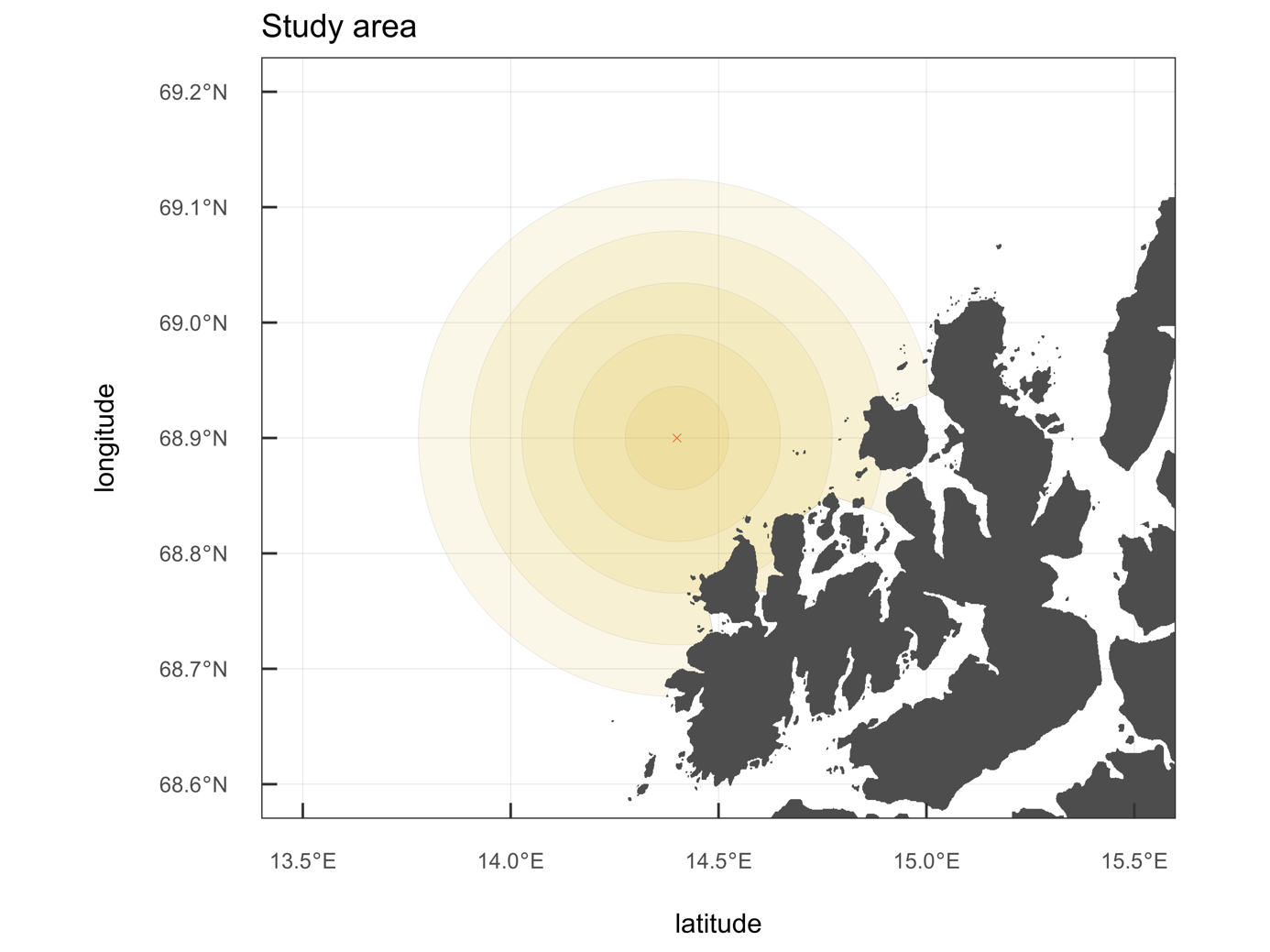


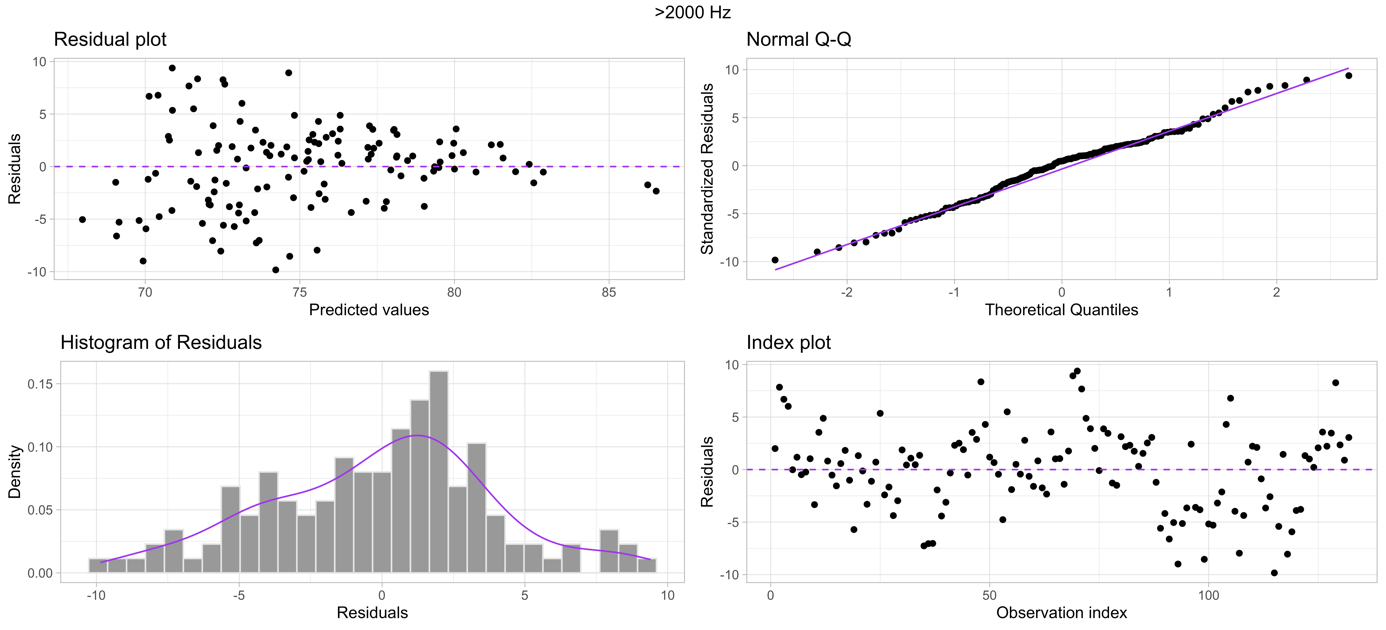

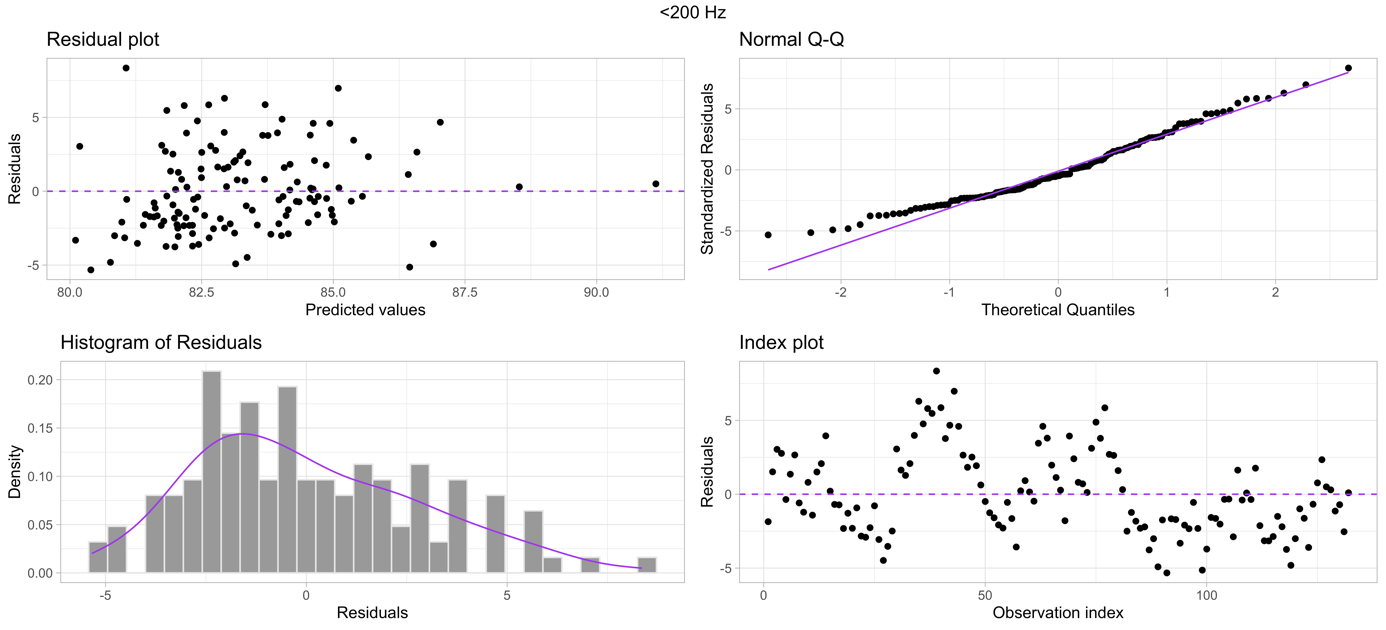

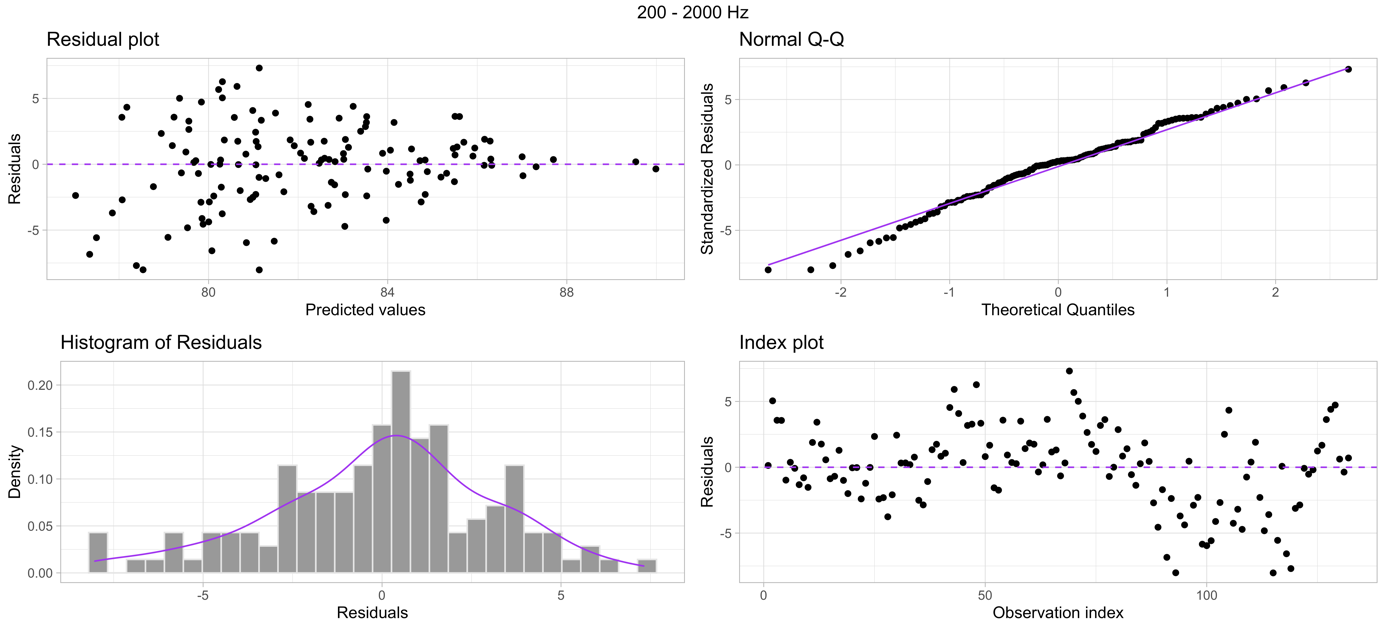
S2: Model diagnostics plots for the 3 selected models, representing the contributions to the soundscape for frequencies below 200 Hz, between 200 Hz and 2 kHz, and above 2 kHz.

S3: Model selection tables for each of the three frequency bins assessed (< 200 Hz, 200 Hz – 2 kHz, and > 2 kHz).

| < 200 Hz | | | | | | | | |
| --- | --- | --- | --- | --- | --- | --- | --- | --- |
|  | Modnames | K | AIC | Delta_AIC | ModelLik | AICWt | Res.LL | Cum.Wt |
| 2 | Ws + sail_010km_All | 5 | 627.8926 | 0.000000 | 1.0000000 | 0.9439464 | -308.9463 | 0.9439464 |
| 3 | Ws + sail_015km_All | 5 | 634.8807 | 6.988129 | 0.0303771 | 0.0286744 | -312.4403 | 0.9726208 |
| 1 | Ws + sail_05km_All | 5 | 635.4535 | 7.560976 | 0.0228116 | 0.0215329 | -312.7268 | 0.9941537 |
| 4 | Ws + sail_020km_All | 5 | 638.4620 | 10.569400 | 0.0050686 | 0.0047844 | -314.2310 | 0.9989382 |
| 5 | Ws + sail_025km_All | 5 | 641.4727 | 13.580138 | 0.0011249 | 0.0010618 | -315.7364 | 1.0000000 |

| < 200 kHz | | | | | | | | |
| --- | --- | --- | --- | --- | --- | --- | --- | --- |
|  | Modnames | K | AIC | Delta_AIC | ModelLik | AICWt | Res.LL | Cum.Wt |
| 2 | Ws + sail_10km_All | 5 | 634.6039 | 0.0000000 | 1.0000000 | 0.6046386 | -312.3019 | 0.6046386 |
| 1 | Ws + sail_05km_All | 5 | 635.4535 | 0.8496616 | 0.6538804 | 0.3953614 | -312.7268 | 1.0000000 |

| < 200 kHz | | | | | | | | |
| --- | --- | --- | --- | --- | --- | --- | --- | --- |
|  | Modnames | K | AIC | Delta_AIC | ModelLik | AICWt | Res.LL | Cum.Wt |
| 6 | Ws + sail_10km_fishing + sail_10km_passenger + sail_10km_cargo + sail_10km_tanker + sail_10km_other | 9 | 622.0423 | 0.00000 | 1.0000000 | 0.9988902 | -302.0211 | 0.9988902 |
| 5 | Ws + sail_10km_other | 5 | 636.8000 | 14.75776 | 0.0006243 | 0.0006236 | -313.4000 | 0.9995138 |
| 3 | Ws + sail_10km_cargo | 5 | 637.3994 | 15.35711 | 0.0004626 | 0.0004621 | -313.6997 | 0.9999759 |
| 1 | Ws + sail_10km_fishing | 5 | 643.6380 | 21.59575 | 0.0000204 | 0.0000204 | -316.8190 | 0.9999964 |
| 2 | Ws + sail_10km_passenger | 5 | 647.8702 | 25.82797 | 0.0000025 | 0.0000025 | -318.9351 | 0.9999988 |
| 4 | Ws + sail_10km_tanker | 5 | 649.3427 | 27.30048 | 0.0000012 | 0.0000012 | -319.6714 | 1.0000000 |

| 200 Hz - 2 kHz | | | | | | | |
| --- | --- | --- | --- | --- | --- | --- | --- |
| Modnames | K | AIC | Delta_AIC | ModelLik | AICWt | Res.LL | Cum.Wt |
| Ws + sail_05km_All | 5 | 666.0210 | 0.000000 | 1.0000000 | 0.5952912 | -328.0105 | 0.5952912 |
| Ws + sail_010km_All | 5 | 667.7258 | 1.704831 | 0.4263838 | 0.2538225 | -328.8629 | 0.8491137 |
| Ws + sail_015km_All | 5 | 669.5054 | 3.484366 | 0.1751377 | 0.1042579 | -329.7527 | 0.9533716 |
| Ws + sail_020km_All | 5 | 671.9961 | 5.975086 | 0.0504112 | 0.0300093 | -330.9981 | 0.9833810 |
| Ws + sail_025km_All | 5 | 673.1780 | 7.157002 | 0.0279175 | 0.0166190 | -331.5890 | 1.0000000 |

| 200 Hz - 2 kHz | | | | | | | | |
| --- | --- | --- | --- | --- | --- | --- | --- | --- |
|  | Modnames | K | AIC | Delta_AIC | ModelLik | AICWt | Res.LL | Cum.Wt |
| 3 | Ws + sail_05km_cargo | 5 | 665.2178 | 0.000000 | 1.0000000 | 0.7690215 | -327.6089 | 0.7690215 |
| 6 | Ws + sail_05km_fishing + sail_05km_passenger + sail_05km_cargo + sail_05km_tanker + sail_05km_other | 9 | 668.3887 | 3.170914 | 0.2048541 | 0.1575372 | -325.1943 | 0.9265587 |
| 2 | Ws + sail_05km_passenger | 5 | 671.5586 | 6.340872 | 0.0419853 | 0.0322876 | -330.7793 | 0.9588463 |
| 1 | Ws + sail_05km_fishing | 5 | 672.3037 | 7.085957 | 0.0289270 | 0.0222455 | -331.1519 | 0.9810918 |
| 5 | Ws + sail_05km_other | 5 | 673.4211 | 8.203396 | 0.0165446 | 0.0127231 | -331.7106 | 0.9938150 |
| 4 | Ws + sail_05km_tanker | 5 | 674.8637 | 9.645972 | 0.0080427 | 0.0061850 | -332.4319 | 1.0000000 |

| > 2 kHz | | | | | | | |
| --- | --- | --- | --- | --- | --- | --- | --- |
| Modnames | K | AIC | Delta_AIC | ModelLik | AICWt | Res.LL | Cum.Wt |
| Ws + sail_05km_All | 5 | 716.9555 | 0.000000 | 1.0000000 | 0.5116198 | -353.4778 | 0.5116198 |
| Ws + sail_010km_All | 5 | 719.0666 | 2.111091 | 0.3480026 | 0.1780450 | -354.5333 | 0.6896648 |
| Ws + sail_015km_All | 5 | 719.6298 | 2.674218 | 0.2626037 | 0.1343533 | -354.8149 | 0.8240181 |
| Ws + sail_020km_All | 5 | 720.3106 | 3.355046 | 0.1868362 | 0.0955891 | -355.1553 | 0.9196072 |
| Ws + sail_025km_All | 5 | 720.6569 | 3.701314 | 0.1571339 | 0.0803928 | -355.3284 | 1.0000000 |

| > 2 kHz | | | | | | | | |
| --- | --- | --- | --- | --- | --- | --- | --- | --- |
|  | Modnames | K | AIC | Delta_AIC | ModelLik | AICWt | Res.LL | Cum.Wt |
| 3 | Ws + sail_05km_cargo | 5 | 714.4924 | 0.000000 | 1.0000000 | 0.5603476 | -352.2462 | 0.5603476 |
| 2 | Ws + sail_05km_passenger | 5 | 716.5931 | 2.100718 | 0.3498121 | 0.1960164 | -353.2965 | 0.7563640 |
| 1 | Ws + sail_05km_fishing | 5 | 718.4455 | 3.953118 | 0.1385452 | 0.0776335 | -354.2227 | 0.8339975 |
| 5 | Ws + sail_05km_other | 5 | 718.4728 | 3.980463 | 0.1366638 | 0.0765792 | -354.2364 | 0.9105767 |
| 4 | Ws + sail_05km_tanker | 5 | 719.0365 | 4.544088 | 0.1031012 | 0.0577725 | -354.5182 | 0.9683492 |
| 6 | Ws + sail_05km_fishing + sail_05km_passenger + sail_05km_cargo + sail_05km_tanker + sail_05km_other | 9 | 720.2400 | 5.747591 | 0.0564841 | 0.0316508 | -351.1200 | 1.0000000 |

# Legends supplementary material

S1: Map of the sampling area for the vessel sailing time dataset. The marked cross represents the location of the acoustic recording station of the LoVe Ocean Observatory, with the distance radii used in estimates of effects of sailing time on sound levels (0-5 km, 5-10 km, 10-15 km, 15-20 km, 20 -25 km). The figure was made using the ggplot ^93^ R ^87^ package. High resolution geography data was acquired through the GSHHG (Global Self-consistent, Hierarchical, High-resolution Geography) database ^98^.

S2: Model diagnostics plots for the 3 selected models, representing the contributions to the soundscape for frequencies below 200 Hz, between 200 Hz and 2 kHz, and above 2 kHz.

S3: Model selection tables for each of the three frequency bins assessed (< 200 Hz, 200 Hz – 2 kHz, and > 2 kHz).
